# 

**Published:** 1916-07

**Authors:** 


					﻿70th ANNIVERSARY YEAR
THE
DENTAL
REGISTER
Edited by Dr NELVILLE S. HOFF
ANN ARBOR, MICH.
VOL. LXX JULY, 1916 NO. 7
THE SAMUEL A. CROCKER CO., Publishers
OHIO DENTAL AND SURGICAL DEPOT
CONRAD E^LDG., 18 & 20 W. Seventh St., CINCINNATI, O.
PRICE, ONE DOLLAR A YEAR
Entered at the Post-Office at Cincinnati, O , as second-class mall matter
				

